# Biomarker Exploration in Human Peripheral Blood Mononuclear Cells for Monitoring Sulforaphane Treatment Responses in Autism Spectrum Disorder

**DOI:** 10.1038/s41598-020-62714-4

**Published:** 2020-04-02

**Authors:** Hua Liu, Andrew W. Zimmerman, Kanwaljit Singh, Susan L. Connors, Eileen Diggins, Katherine K. Stephenson, Albena T. Dinkova-Kostova, Jed W. Fahey

**Affiliations:** 1 Department of Pharmacology and Molecular Sciences, Johns Hopkins University School of Medicine, Baltimore, Maryland United States of America; 2 Cullman Chemoprotection Center, Johns Hopkins University, Baltimore, Maryland United States of America; 3 Department of Pediatrics, University of Massachusetts Medical School, Worcester, Massachusetts, United States of America; 4 Department of Neurology, University of Massachusetts Medical School, Worcester, Massachusetts, United States of America; 5 Jacqui Wood Cancer Centre, Division of Cellular Medicine, Ninewells Hospital and Medical School, University of Dundee, Dundee, United Kingdom; 6 Department of Medicine, Johns Hopkins University School of Medicine, Baltimore, Maryland United States of America; 7 Department of International Health, Johns Hopkins University Bloomberg School of Public Health, Baltimore, Maryland United States of America

**Keywords:** Biomarkers, Diseases, Medical research

## Abstract

Autism Spectrum Disorder (ASD) is one of the most common neurodevelopmental disorders with no drugs treating the core symptoms and no validated biomarkers for clinical use. The multi-functional phytochemical sulforaphane affects many of the biochemical abnormalities associated with ASD. We investigated potential molecular markers from three ASD-associated physiological pathways that can be affected by sulforaphane: redox metabolism/oxidative stress; heat shock response; and immune dysregulation/inflammation, in peripheral blood mononuclear cells (PBMCs) from healthy donors and patients with ASD. We first analyzed the mRNA levels of selected molecular markers in response to sulforaphane *ex vivo* treatment in PBMCs from healthy donors by real-time quantitative PCR. All of the tested markers showed quantifiability, accuracy and reproducibility. We then compared the expression levels of those markers in PBMCs taken from ASD patients in response to orally-delivered sulforaphane. The mRNA levels of cytoprotective enzymes (NQO1, HO-1, AKR1C1), and heat shock proteins (HSP27 and HSP70), increased. Conversely, mRNA levels of pro-inflammatory markers (IL-6, IL-1β, COX-2 and TNF-α) decreased. Individually none is sufficiently specific or sensitive, but when grouped by function as two panels, these biomarkers show promise for monitoring pharmacodynamic responses to sulforaphane in both healthy and autistic humans, and providing guidance for biomedical interventions.

## Introduction

Autism Spectrum Disorder (ASD) is one of the most common neurodevelopmental disorders that, in the United States, is currently estimated to affect 1 out of 59 children who are 8 years old^1^. Despite decades of research and advances in our knowledge of the etiologies of ASD, treatments and biomarkers for ASD remain limited^2–5^. To date, the primary diagnosis of ASD still relies on observational tools that are by nature subjective^3,6^. Genetic and metabolic studies may provide additional clues to specific etiologies or treatments, but there are no generally accepted biological markers for commonly affected cellular pathways in ASD^5,6^.

Studies over the last two decades have implicated that physiological and metabolic abnormalities, such as immune dysregulation/neuro-inflammation, redox imbalance/oxidative stress, mitochondrial dysfunction, environmental toxicant exposures and gut dysbiosis, are important aspects of the pathophysiology of ASD^6^. Closer examination of the biological markers of pathways associated with ASD could be informative regarding its pathophysiology, and might be useful for early identification, prognosis and treatment. Most importantly, they might guide treatment strategies and enable clinicians to monitor treatment responses^3,7^.

Since ASD is multi-factorial and multiple genes have been implicated with no specific drug targets, strategies using multi-functional phytochemicals are highly attractive. Sulforaphane [1-isothiocyanato-4-(methylsulfinyl)-butane, SF] is a dietary phytochemical, derived from its biologically inactive precursor glucoraphanin (GR) that is widely consumed in the edible cruciferous plant, broccoli (*Brassica oleracea* var. *italica*). SF is produced by the action of the enzyme myrosinase, which is present in cruciferous plant cells and is normally segregated from GR until the cells are ruptured^8,9^. Myrosinase is also produced by the microflora of the gastrointestinal tract of all healthy humans^10,11^. For over two decades, evidence of the beneficial effects of SF has accumulated, extending from *in vitro* studies, to animal models, to a variety of clinical studies^6,12–15^. Importantly, there are currently no drugs approved to treat the core symptoms of ASD, nor are there any studies using SF in genetic mouse models of ASD^16^. The cytoprotective potential of SF extending even to maternal dietary supplementation to prevent perinatal brain injury has been considered^17^. In our previous placebo-controlled, double-blinded, randomized clinical trial, daily administration of SF for 4-18 weeks substantially improved the behavioral abnormalities of the majority of 26 young males with moderate to severe ASD without significant toxicity^18,19^.

**Figure 1:**
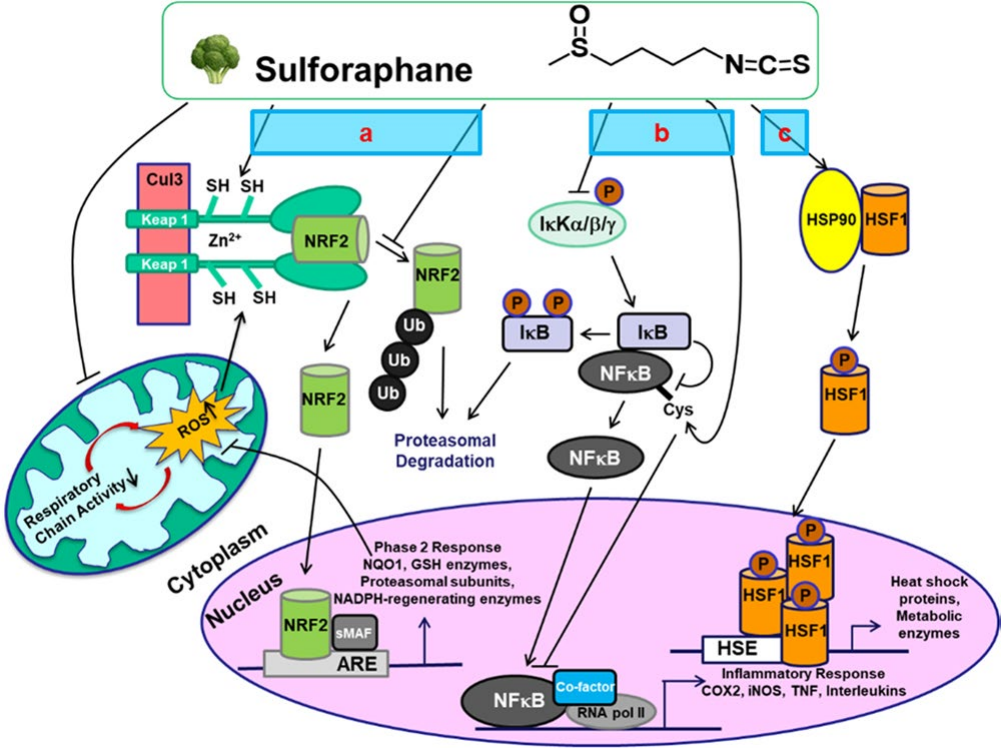
Major signaling pathways for protective mechanisms against ASD by SF. (**a**) Keap1/Nrf2/ARE pathway, (**b**) NF-κB inflammatory pathway, (**c**) heat-shock responses. (modified from Liu *et al*.^6^).

SF is a multi-functional phytochemical that acts upon many of the same biochemical and molecular (biomarker) pathways in which abnormalities have been ascribed to ASD, including oxidative stress, mitochondrial dysfunction, and neuroinflammation^6,20–27^. SF is one of the most potent naturally occurring inducers of mammalian cytoprotective enzymes through the Kelch-like ECH associated protein 1 (Keap1)/Nuclear factor erythroid 2-related factor 2 (Nrf2)/antioxidant response element (ARE) signaling pathway (Fig. 1). Nrf2 activation is increasingly understood to play a significant role in ameliorating many diseases, including neurological disorders^28^. This is in part a consequence of the recognized role of oxidative stress and chronic inflammation as causative factors for these disorders, and Nrf2 is the master regulator of cellular redox homeostasis and an inhibitor of inflammation, both of which are critical factors in the neuropathology of ASD^29,30^. The brain is particularly vulnerable to oxidative stress because of its high oxygen consumption, high content of unsaturated fatty acids and transition metals, and low antioxidant defense capacities^31,32^. SF has also shown strong anti-inflammatory activity in various settings, including animal models of neuroinflammation^25,33,34^. The anti-inflammatory activity of SF is partially Nrf2-dependent^35^ and is additionally mediated through inhibition of the nuclear factor-κB (NF-κB) pathway (Fig. 1), resulting in decreased expression of many cytokines and other pro-inflammatory factors^36,37^. Nrf2 itself also exhibits robust anti-inflammatory activity through at least three independent and critical mechanisms: modulation of redox metabolism; crosstalk with NF-κB; and direct negative regulation of proinflammatory genes^6,26,32,35,38–40^.

Although the widespread anecdotal reports of the fever response in a substantial fraction of autistic patients have been corroborated^41,42^, the mechanisms of the “fever effect” in patients with ASD are just beginning to be understood^43^. Fever may activate general cellular stress responses, which involve at least two cellular signaling pathways: the Keap1/Nrf2/ARE cytoprotective pathway and the heat shock/proteasomal pathway that protects against a wide variety of disturbances of cellular functions, and these two pathways are interrelated^44,45^. SF has been shown to be an efficient inducer of a number of heat shock proteins (HSPs) in several human cell lines^46–48^, suggesting that activation of the heat shock response could be another contributing factor to SF-mediated protection in ASD.

In search for biomarkers for monitoring responses to SF treatment in ASD, we investigated several potential molecular markers from three ASD-associated basic physiological pathways that can be affected by SF (Fig. 1a–c): (1) redox metabolism/oxidative stress, (2) heat shock response, and (3) immune dysregulation/inflammation, in peripheral blood mononuclear cells (PBMCs). To optimize the methodology, we first isolated PBMCs from fresh human blood from healthy donors, and exposed these cells to SF *ex vivo*. We then examined a subset of these markers in PBMCs from blood taken from subjects with ASD before and after 14 d of daily ingestion of a SF-providing nutritional supplement, calculated to deliver approximately the same daily dose of SF as was provided in our previous study^18^. Criteria evaluated for the biomarkers in these PBMCs included quantifiability, accuracy and reproducibility of the assay methods, as well as sensitivity and responsiveness to both *ex vivo* and *in vivo* SF treatments.

## Results

### Sulforaphane induced cytoprotective gene expression in human PBMCs with *ex vivo* treatment

There is a long history of studies showing that ASD is associated with oxidative stress and diminished antioxidant capacity^49–51^. Eukaryotic organisms have developed highly efficient protective mechanisms, which do not normally operate at their maximal capacity, but can be induced to maintain cellular redox homeostasis and to reduce oxidative stress by production of direct antioxidants, and by induction of detoxifying enzymes, primarily through the Keap1/Nrf2/ARE pathway (Fig. 1)^21,52,53^. Much evidence also points to the neuroprotective role of HSPs, and the enhanced susceptibility of cells to damage when HSPs are suppressed^54^.

**Figure 2:**
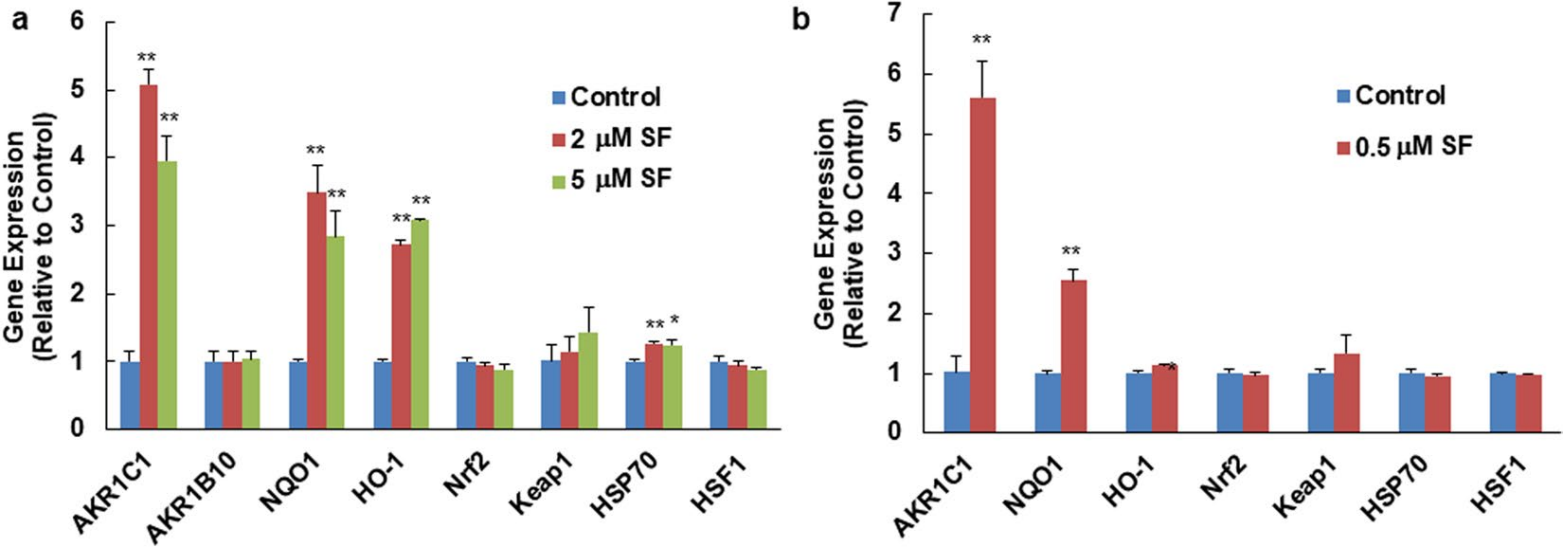
Cytoprotective gene expression in human PBMCs from healthy donors in response to SF *ex vivo* treatment. (**a**) Single dose. (**b**) Three consecutive, and lower daily doses. In each case assessment was made 6 hours following the final dose. Means ± SD are shown. Symbols *(*P* < 0.05) or **(*P* < 0.01) indicate statistical differences between treatments and controls by two-tailed Student’s *t*-test.

To evaluate the potential of using the expression levels of some of these cytoprotective genes as biomarkers for ASD treatment with SF, we first analyzed the mRNA levels of selected molecular markers of the Keap1/Nrf2/ARE pathway and the heat shock pathway in response to SF *ex vivo* treatment in human PBMCs isolated from healthy donors. Expression of Keap1/Nrf2/ARE pathway-related markers, including Nrf2, Keap1, NAD(P)H: quinone oxidoreductase-1 (NQO1), heme oxygenase-1 (HO-1), and aldo-keto reductases (AKR1C1 and AKR1B10), were analyzed by quantitative real-time PCR. NQO1, HO-1 and AKR1C1 were significantly induced by 6-h SF treatment. This time point was chosen based on our earlier observations of the kinetics of upregulation of Nrf2-dependent genes by SF, and was expected to capture the increased mRNA production of both very fast (HO-1) and relatively slow (NQO1) responders^55^. Interestingly, AKR1C1 was the most highly inducible Nrf2-target gene (up to a 5-fold increase), but the functionally-related AKR1B10 was not inducible. The mRNA levels for Keap1 increased slightly, and those for Nrf2 did not change significantly in response to SF treatment (Fig. 2a).

Expression of HSP70 and HSF1, two markers of heat shock response, were also analyzed in PBMCs by real-time PCR. HSP70 was slightly but significantly elevated by SF treatment, whereas HSF1 did not change (Fig. 2a).

Notably, there was no concentration-dependence in the induction of any of the genes examined, with the higher (5 μM) concentration of SF even showing a slightly diminished effect for the induction of AKR1C1 and NQO1. Although this concentration is achievable *in vivo*^55^, more typical peak concentrations of SF (and its metabolites) in human plasma are 1-2 μM^56^. In order to evaluate whether PBMCs would respond to repeated, lower dose SF *ex vivo* treatment, isolated PBMCs from healthy donors were exposed to vehicle (0.1% acetonitrile) or low concentration SF (0.5 μM) repeatedly, every 24 hours for 3 consecutive days, which we felt would be more relevant to the clinical condition, although SF metabolites in this scenario might not be identical to *in vivo* treatment. Six hours after the last treatment, cells were collected and the expression of the same markers was analyzed. AKR1C1 was significantly induced by this multiple low dose SF treatment to similar extent as by a single higher dose SF treatment, while NQO1 was significantly, but less robustly induced by this lower concentration of SF (Fig. 2b). None of the other genes that we measured were significantly altered by repeated low dose SF treatments (Fig. 2b).

### Sulforaphane decreased pro-inflammatory gene expression stimulated by LPS in human PBMCs with *ex vivo* treatment

**Figure 3:**
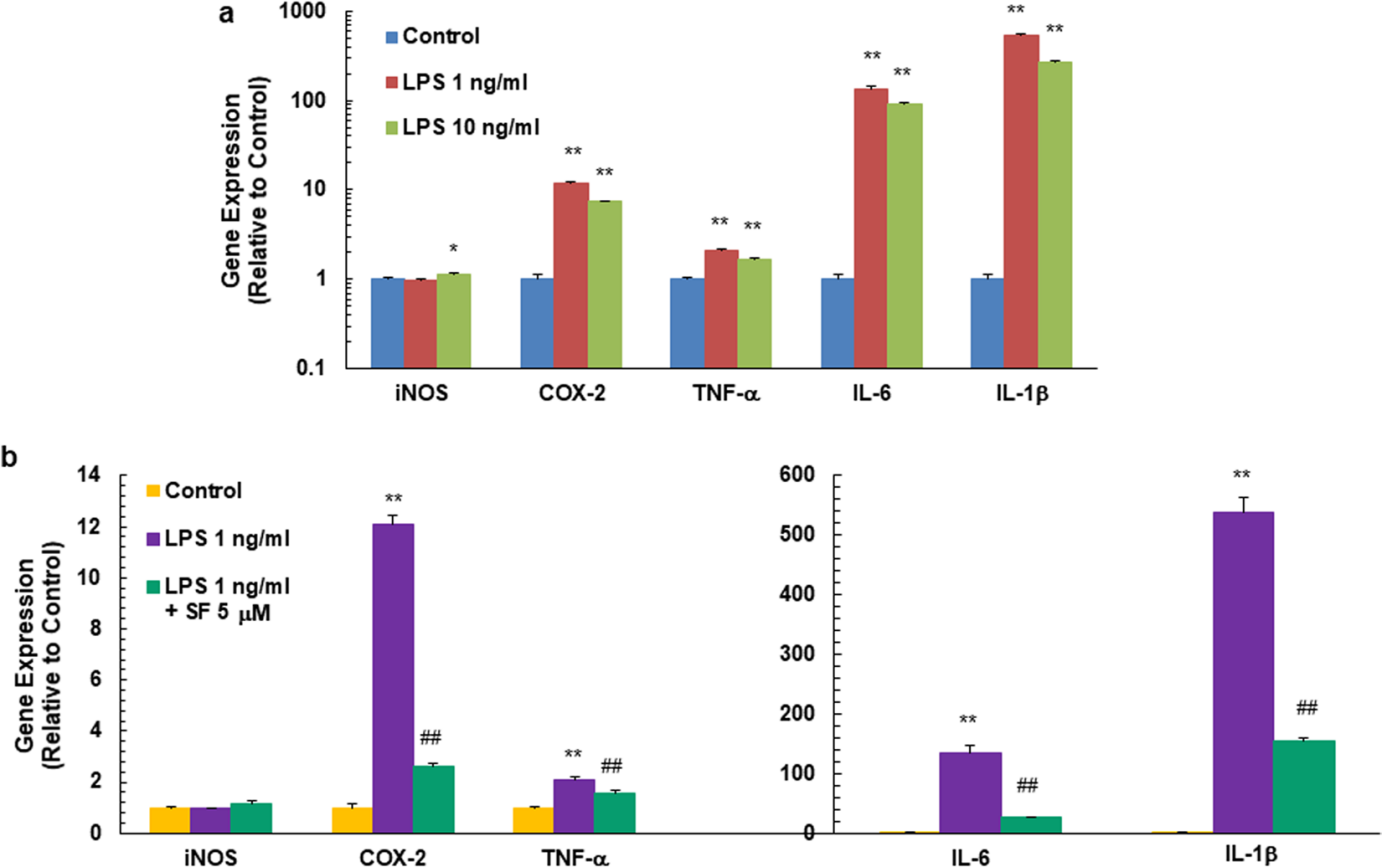
Pro-inflammatory gene expression in human PBMCs from healthy donors in response to LPS stimulation (**a**) and to SF pre*-*treatment plus LPS stimulation (**b**). Means ± SD are shown. Symbols *(*p* < 0.05) or **(*p* < 0.01) indicate statistical differences between treatments and controls by two-tailed Student’s *t*-test (**a**,**b**), and symbol ^##^(*p* < 0.01) indicates statistical differences between LPS stimulation and SF pre-treatment plus LPS stimulation (**b**).

Inflammation and immune dysregulation have been observed both within the brain and in the periphery in ASD patients^6,51,57–63^. Increased expression of immune-related genes, production of atypical pro-inflammatory cytokines and other inflammatory factors, all lead to a chronic state of inflammation in the central nervous system and in the peripheral immune system of ASD patients^51,64,65^. Expression of pro-inflammatory markers can be up-regulated by inflammatory mediators such as the bacterial endotoxin lipopolysaccharide (LPS), through the I kappa B kinase (IκK)/NF-κB pathway (Fig. 1). In addition, postnatal LPS challenge (at days 5 and 7 after birth) has been reported to cause depressive, anxiety-like, repetitive behavior, and working memory deficits in pre-adolescent (35-day-old) male mice^66^. We therefore treated PBMCs obtained from healthy donors with 1 ng/mL or 10 ng/mL of LPS for 5 hours, following which, expression of pro-inflammatory markers inducible nitric oxide synthase (iNOS), cyclooxygenase-2 (COX-2), tumor necrosis factor-α (TNF-α), interlukin-6 (IL-6) and interlukin-1β (IL-1β) were analyzed by real-time PCR. All inflammatory markers except for iNOS were responsive to the LPS stimulation, especially IL-6 and IL-1β (up to several hundred-fold), and were more responsive to the lower dose of LPS (Fig. 3a). Thirty minutes pre-treatment by SF inhibited the LPS-stimulated up-regulation of COX-2, TNF-α, IL-6 and IL-1β expression up to 80% of control levels (Fig. 3b).

### Biomarkers show consistent responsiveness to SF *ex vivo* treatment over time in human PBMCs

**Figure 4:**
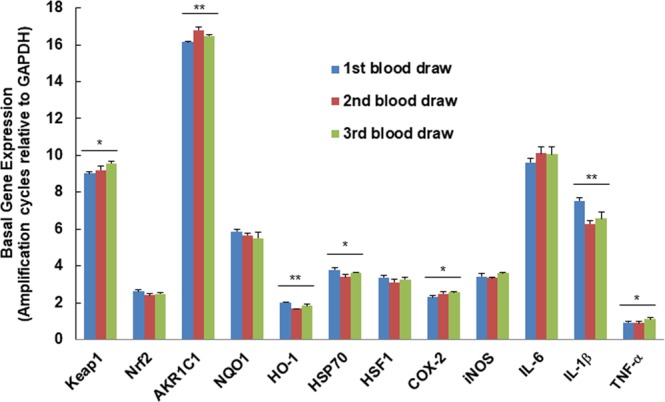
Basal gene expression levels in human PBMCs isolated from three blood samples of the same healthy donor. Means ± SD are shown. Symbols *(*p* < 0.05) or **(*p* < 0.01) indicate statistical differences among three blood draws by one-way ANOVA.

**Figure 5:**
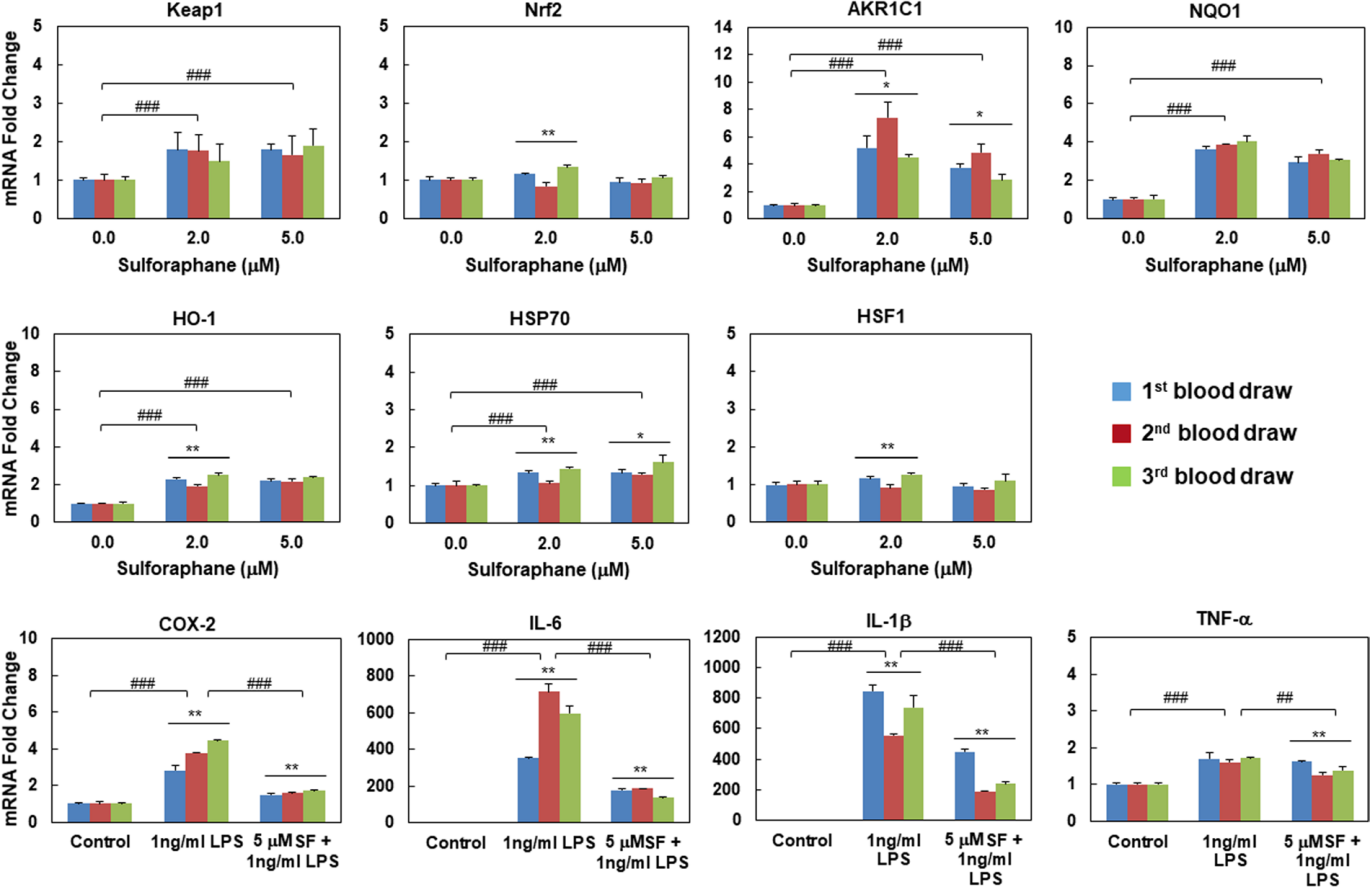
Consistency of gene expression in human PBMCs from healthy donors in response to *ex vivo* treatment among three blood samples of the same healthy donor. Means ± SD are shown. Symbols *(*p* < 0.05) or **(*p* < 0.01) indicate statistical differences among three blood draws by one-way ANOVA, and symbols ^##^(*p* < 0.01) or ^###^(*p* < 0.001) indicate statistical differences between treatments by two-tailed Student’s *t*-test.

In order to evaluate the consistency of the above-analyzed markers, PBMCs were isolated from fresh human blood drawn for three consecutive weeks from the same healthy donor. Basal gene expression levels were analyzed in these PBMCs. Although there were differences in the mRNA levels for a few markers among sets of three consecutive blood draws from the same donor (Fig. 4), these differences were negligible when compared to the fold changes of these markers after stimulation (Fig. 5).

A portion of the PBMCs from the three blood draws were identically treated with vehicle (0.1% acetonitrile) or SF for 6 h, and cytoprotective markers of the Keap1/Nrf2/ARE pathway and the heat shock response were compared. In a separate experimental setting, PBMCs isolated from the above mentioned three blood draws were identically treated with SF for 30 min and then LPS for an additional 5 h, followed by the comparison of pro-inflammatory markers. Although there were differences among the three repeated blood draws for most of the markers (indicated by * or ** in Fig. 5), these differences were negligible when compared to the differences of these markers between pre- and post-treatment (indicated by ^##^ or ^###^ in Fig. 5).

### Biomarker evaluation in PBMCs from ASD patients with *in vivo* SF treatment

As a pilot study for a clinical trial of SF in children with ASD (*clinicaltrials.gov* NCT02561481), we evaluated the same biomarkers from the *ex vivo* studies in 10 young males with ASD, 6-12 years of age (Table 1), who received SF (in the form of a dietary supplement containing GR and myrosinase), 2.2 μmol/kg/d for 14 days. We chose this dietary supplement, because it represents a highly standardized commercially available source of SF that could be easily obtained by patients and caregivers. Blood and urine samples were collected before and at the end of treatment. This study design allowed for each patient to serve as his own control. Notably, a placebo group was not included for biomarker evaluation in this pilot study, because it is difficult to subject ASD children to blood draws, and there are no endogenous sources of SF in human tissues, as we have shown in a previous placebo-controlled study^67^. There were no serious adverse events; one was judged by his parents to be more hyperactive, one had increased urinary frequency, two had increased flatus, one had nausea, and one complained of unpleasant taste. No clinical changes were reported by the families of 8 of the 10 participants. The parents of one child reported increased eye contact and sound sleep, another had improved behavior.

**Table 1:** Sulforaphane bioavailability as a function of treatment.

Subjects*	Age	Pre-Dose DTC	% Excretion	Plasma DTC (nmol/mL)		Time between last dose
	(Years)	in urine	in urine	pre-dose	post-dose	and blood draw
1	9	none	54.1	0.004	0.052	15.9
2	10	none	16.1	0.006	0.159	8.9
3	6	none	10.3	0.002	0.088	7.5
4	12	none	52.4	0.026	0.257	9.7
5	9	none	68.4	0.007	0.307	9.4
6	10	none	8.2	0.008	0.014	19.1
7	10	none	30.4	0.011	0.13	7.25
8	12	none	37.2	0.019	0.016	1.9
9	9	none	37.6	0.047	0.09	1.75
10	9	none	29.4	0.003	0.16	14

*All subjects are males.

We used excretion of total isothiocyanates (ITC) and their metabolites (dithiocarbamates, DTC) in urine as a proxy for the bioavailability of GR delivered. Timed urine collection at the end of 14 days were used to determine excretion of SF and its metabolites as DTC by cyclocondensation reaction-HPLC assay. DTC levels were also measured in the plasma. The non-detectable or extremely low pre-dose DTC levels in both urine and plasma samples indicated that, as instructed, no patient was consuming a diet or supplements containing glucosinolates or ITC, including SF (Table 1). Conversion of GR to SF (bioavailability) was calculated as percent of dose excreted in collected urine samples, and was normalized on a molar equivalence basis (moles of GR to moles of SF plus its metabolites as determined in the cyclocondensation reaction). Conversion efficiencies varied among subjects from 8.2% to 68.4% of the administered dose, which is consistent with the data we and others have previously reported on bioavailability in adults^11,68,69^. Post-dose plasma DTC levels were closely related to the time between last dose and blood draw, with lower levels at less than 2 h or more than 14 h, and higher levels between 9-10 h after the last dose (Table 1). In addition, the plasma DTC value for each patient was well correlated with the conversion efficiency calculated in his urine sample, taking the time between last dose and blood draw into consideration.

Using PBMCs isolated from blood samples of six of the ten ASD subjects in the pilot study, we compared the expression levels of 9 molecular markers related to our hypothesized mechanisms of the action of SF. These markers were selected in large part based on their responsiveness to SF *ex vivo* treatment, with (a) AKR1C1, NQO1, HO-1 serving as markers for the Keap1/Nrf2/ARE cytoprotective pathway; (b) COX-2, TNF-α, IL-6 and IL-1β serving as markers for the inflammatory pathway; and (c) HSP70 and HSP27 serving as markers for the heat shock response.

**Figure 6:**
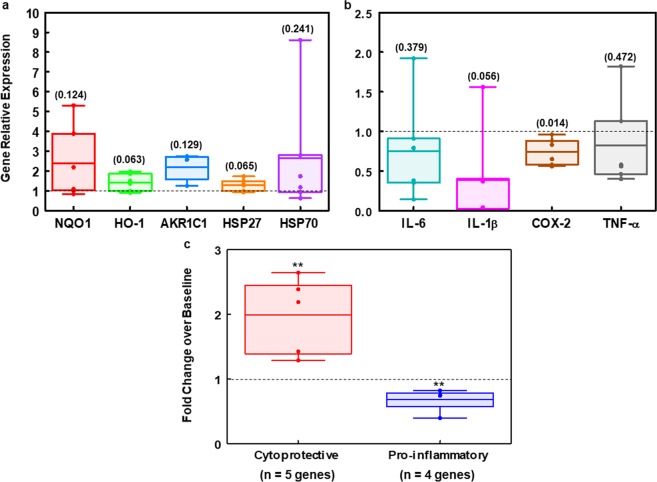
Gene expression level changes in PBMCs from 6 patients with ASD after SF intervention. SF was orally administered daily at the dose of 2.2 μmol/kg body weight in the form of GR plus myrosinase for two weeks. Expression level of each gene immediately prior to treatment was set as control. (**a**) Cytoprotective genes. (**b**) Pro-inflammatory genes. (**c**) Grouped genes by broad functionality (e.g. cytoprotective or pro-inflammatory). Differences between pre-dose and post-dose for individual markers were analyzed by two-tailed paired *t*-test, and *p*-values for each marker are the numbers in parentheses above the top error bars (**a**,**b**). Differences of grouped markers from baseline were analyzed by one-way ANOVA, and symbol ** indicates highly significant statistical difference (*p* < 0.01) (**c**). Data points for each subject (**a**,**b**) or me**a**ns of each group of genes (**c**) are plotted. Horizontal lines within boxes are means.

Overall, the magnitude of change of all markers examined was smaller in the *in vivo* than it was in the *ex vivo* treatments. Although there was considerable variability among subjects, after 2 weeks of oral administration of this dietary supplement, the expression of the Nrf2-target genes NQO1, HO-1, and AKR1C1, as well as the heat shock response markers HSP27 and HSP70, were elevated in PBMCs from patients with ASD (Fig. 6a). Conversely, the levels of pro-inflammatory markers (IL-6, IL-1β, COX-2 and TNF-α) decreased (Fig. 6b). When differences between pre-dose (relative expression set at 1.0) and post-dose for all the tested markers were analyzed individually by two-tailed paired *t*-test, only the differences in COX-2 expression were significant (*p* = 0.014). However, grouping by broad functionality (e.g. cytoprotective or pro-inflammatory), differences from baseline were highly significant at the 95% confidence level by ANOVA (F_1,52_ = 8.82, *p* < 0.005 for cytoprotective genes and F_1,46_ = 9.25, *p* < 0.004 for pro-inflammatory genes) (Fig. 6c).

## Discussion

Development of reliable ASD biomarkers is critical for elucidating ASD pathobiology, improving diagnosis and monitoring therapeutic effects. The brain is the central affected organ in ASD, despite significant comorbidities in other systems, notably the gastrointestinal tract. Studies examining neural tissue are, therefore, likely to be more informative than analyses of other tissues in terms of understanding the pathogenesis of brain dysfunction in ASD. However, brain biopsy- or cerebrospinal fluid-based biomarkers are invasive, MRI and PET-based imaging biomarkers are expensive and difficult to perform, which limits their appeal to both patients and physicians. There is now growing interest in identification of molecular biomarkers that are less- or non-invasive, cost-effective, and readily available in clinical settings. Therefore, reliable blood-based biomarkers are considered essential for ASD.

We used human PBMCs isolated from blood of healthy donors and patients with ASD to explore potential biomarkers in response to SF in ASD. Expression of markers of three signaling pathways related to ASD pathophysiology were evaluated by quantitative real-time PCR. Three representative Nrf2-dependent enzymes, AKR1C1, NQO1 and HO-1, were significantly induced by 6 h of 2 μM or 5 μM SF *ex vivo* treatments in PBMCs from healthy donors. However, AKR1B10 was not induced. In the hands of other investigators, the AKR1 subfamily of aldo-keto reductases was the most highly upregulated family of genes through the Keap1/Nrf2 pathway in human gut mucosa and different cell lines^70–72^; these genes were thus thought to be good biomarker candidates. We chose AKR1C1 and AKR1B10 as Nrf2-dependent markers in our study since they were highly responsive to SF treatment in human breast epithelial cells and keratinocytes^71,72^. Notably however, although AKR1C1 and AKR1B10 share Nrf2 as a transcriptional regulator, AKR1B10 is also regulated by p53^73^. AKR1C1 and NQO1, but not HO-1, were also induced by a lower dose of SF (0.5 μM) over a 3-day treatment. Although the heat shock response marker HSP70 was not very responsive to the SF concentrations we used for the *ex vivo* treatment, the change was statistically significant following treatment for 6 h with 2 or 5 μM SF. We also evaluated expression of the transcription factors of the above two pathways, Nrf2 and HSF1, in response to SF treatment in PBMCs from healthy donors. Nrf2 is primarily regulated at the level of its protein stability, whereas HSF1 is mainly regulated by post-translational modifications. As expected, their expression levels were not responsive to SF *ex vivo* treatment. However, comparing their basal expression levels in PBMCs from ASD patients to age-matched healthy controls may provide valuable information on pathological mechanisms of ASD. An earlier clinical study showed that Nrf2 mRNA levels are substantially depressed in granulocytes of ASD children (45% of the levels in typically developing children)^74^. Keap1, as the cytoplasmic regulator of Nrf2 activity, had slightly increased expression following SF treatment, however, SF induces the Nrf2-dependent enzymes mainly through modifying specific and highly reactive cysteine residues of Keap1 and not by changing its expression^13,75^. Moreover, SF *ex vivo* pre-treatment significantly decreased the LPS-stimulated inflammatory gene (COX-2, TNF-α, IL-6 and IL-1β) expression levels in PBMCs from healthy donors.

Although variable basal gene expression levels are manifest in PBMCs of different blood donors, the trends of their responsiveness to treatment were similar. More importantly, basal gene expression of our tested markers in PBMCs from different blood draws from the same donor were very similar, and the differences in responsiveness to treatment among the PBMCs from different blood draws were negligible when compared to the fold changes of these markers in response to treatments. Therefore, these markers have the potential to serve as biomarker candidates.

This study represents our attempt to develop biomarkers and explore molecular basis of the treatment effects of SF on patients with ASD. The *in vivo* evaluation of selected markers is a pilot study for a clinical trial of SF in children with ASD (*clinicaltrials.gov* NCT02561481). By executing this pilot study, we expected to gather information on the reproducibility of individual biomarker values and magnitudes of changes resulting from short term SF intervention and to identify the precise biomarker endpoints that would be measured in the main clinical trial. Although we collected urine and blood samples from 10 subjects, unfortunately, the RNA quality from PBMCs of the first 3 participants was compromised due to inappropriate processing and storage, and 1 participant had a gastrointestinal infection at the time of the post-treatment blood draw, which limited our *in vivo* biomarker evaluation to samples from 6 participants. Perhaps due to the relatively small sample size, the increased expression of individual cytoprotective markers (NQO1, AKR1C1, HO-1, HSP70 and HSP27) and the decreased expression of most individual pro-inflammatory markers (IL-6, IL-1β and TNF-α), except that of COX-2, after 2 weeks of SF treatment did not reach statistical significance. However, the functionally grouped markers showed great potential as panel biomarkers to guide treatment strategies and enable clinicians to monitor treatment responses. In addition, our pilot study only exposed participants to SF for 2 weeks, which is less than the exposure that resulted in changes in clinical symptoms in our previous study^18^. Longer exposure times may well be necessary, and the correlation between biomarker changes and clinical improvements will be reported from the recently completed full clinical trial.

In conclusion, in the above *ex vivo* and *in vivo* experiments using human PBMCs from healthy donors and patients with ASD, the tested markers show potential as biomarkers for monitoring SF responses in humans. They are quantifiable, and the assay method is accurate and reproducible; the samples are readily obtainable in quantities needed for measurement, and sampling is minimally invasive. Most importantly, the biomarkers show sensitivity and consistent responsiveness to SF treatment both *ex vivo* and *in vivo*. Although as a single marker, none of them is specific or sensitive enough, these biomarkers, grouped by function as two panels, show promise in monitoring responses to treatments, and in providing guidance for the selection and efficacy of biomedical interventions. We conducted this study in the context of ASD, however our findings have broader implications and suggest that these biomarkers can be used in any study involving an intervention with SF.

## Methods

### Participants and treatment

For the *ex vivo* experiments, 7 healthy adult donors were recruited. Blood was drawn a single time from 5 donors. Two of them each had blood drawn weekly, for 3 consecutive weeks. All experiments were performed in accordance with relevant guidelines and regulations at Johns Hopkins University and informed consent was obtained from all participants prior to blood collection, under a research protocol approved by the Institutional Review Board of Johns Hopkins University School of Medicine (NA_00036496).

As a pilot study for a clinical trial of SF in children with ASD (*clinicaltrials.gov* NCT02561481), 10 young males with ASD, 6-12.5 years of age (mean, 9.9 years) were recruited to evaluate potential blood-based biomarkers in response to oral SF treatment. This study was conducted at the UMass Memorial Medical Center and University of Massachusetts Medical School, and Johns Hopkins University with approval from the Institutional Review Boards of both institutions (IRBH00007832 and IRB00084331). The diagnosis of moderate to severe ASD was confirmed by testing using the Autism Diagnostic Observation Schedule (ADOS)^76^ and all subjects had screening medical histories, examinations and normal clinical laboratory screening following parental consent. Each subject received SF in the form of Avmacol tablets (crushable), each containing 12.5-15 mg glucoraphanin (GR, the precursor of SF) and active plant-derived myrosinase (the enzyme that converts GR to SF). Tablets were provided along with a COA, by Nutramax Laboratories, Inc. (Edgewater, Maryland, USA), under an IND from the US FDA. Dosing was calibrated to ca. 2.2 μmol SF/kg body weight per day for 14 days. Subjects’ blood and urine samples were collected before, and at the end of the treatment period. All experiments were performed in accordance with relevant guidelines and regulations at University of Massachusetts and Johns Hopkins University.

### Blood sample collection and PBMC isolation

Eight mL of whole blood were drawn from the participants into Vacutainer CPT tubes (Becton, Dickinson and Company, Franklin Lakes, NJ, USA) at room temperature and processed for PBMC isolation according to the instructions from the manufacturer. Briefly, blood in a CPT tube was centrifuged, PBMCs suspended in a small amount of plasma were transferred to a 50-mL conical tube, and PBMCs were washed twice with phosphate buffered saline. Isolated PBMC pellets from healthy donors were re-suspended in warm RPMI 1640 culture medium with 10% heat-inactivated FBS for *ex vivo* treatment. PBMC pellets isolated from ASD patients were stored at −80 °C for future RNA isolation.

### *Ex vivo* treatment of PBMCs

PBMCs isolated from blood of a healthy donor were plated in 6-well plates (~4 × 10^6^ cells/well) in RPMI 1640 medium plus 10% heat-inactivated FBS. After incubation for 2 h, some of the cells were treated with vehicle (0.1% acetonitrile) or SF (2 μM and 5 μM) for 6 h, or exposed to vehicle (0.1% acetonitrile) or low dose SF (0.5 μM) repeatedly for 3 consecutive days. Meanwhile, some of the PBMCs were exposed to vehicle or SF for 30 min and then lipopolysacharide (LPS) for an additional 5 h to stimulate inflammatory response. After treatments, cells were collected for RNA isolation.

### Total RNA isolation and quantitative real-time PCR

Total cellular RNA was isolated from PBMCs using the RNeasy mini kit (Qiagen, Valencia, CA, USA). RNA quantity and quality were measured with the NanoDrop2000 spectrophotometer (Thermo Fisher Scientific, Waltham, MA, USA) and complementary DNAs (1 µg) were synthesized using the iScript cDNA Synthesis Kit (Bio-Rad Laboratories, Hercules, CA, USA). Quantitative real-time PCR analysis was performed using the Applied Biosystems QuantStudio 3 Real-Time PCR System (Thermo Fisher Scientific, Waltham, MA, USA). All primers were optimized, and a final primer concentration of 300 nM was used for all reactions. Primer sequences for gene amplification are shown in Table 2. The reactions were assembled using 2.5~25 ng of cDNA, 1× PowerUp SYBR Green Master Mix (Applied Biosystems, Thermo Fisher Scientific, Waltham, MA, USA), forward and reverse primers, and nuclease-free water. Relative mRNA expression was normalized to GAPDH. Gene expression was calculated using the comparative 2^−ΔΔCT^ method^77,78^.

**Table 2:** Sequences of real-time PCR primers.

Primers		Sequences
Keap1	forward	5′-GGG TCC CCT ACA GCC AAG-3′
	reverse	5′-TGG GGT TCC AGA AGA TAA GC-3′
Nrf2	forward	5′-ACA CGG TCC ACA GCT CAT C-3′
	reverse	5′-TGC CTC CAA AGT ATG TCA ATC A-3′
NQO1	forward	5′-CAG CTC ACC GAG AGC CTA GT-3′
	reverse	5′-GAG TGA GCC AGT ACG ATC AGT G-3′
AKR1C1	forward	5′-CGC CTG CAG AGG TTC CTA AAA-3′
	reverse	5′-ATC AAT ATG GCG GAA GCC AG-3′
HO-1	forward	5′-GGG TGA TAG AAG AGG CCA AGA-3′
	reverse	5′-AGC TCC TGC AAC TCC TCA AA-3′
HSF1	forward	5′-CAT GAA GCA TGA GAA TGA GGC T-3′
	reverse	5′-ACT GCA CAG TGA GAT CAG GA-3′
HSP70	forward	5′-ATG AGT ATA GCG ACC GCT GC-3′
	reverse	5′-TCC TTG GAC TGT GTT CTT TGC-3′
HSP27	forward	5′-TCC CTG GAT GTC AAC CAC TTC-3′
	reverse	5′-TCT CCA CCA CGC CAT CCT-3′
COX-2	forward	5′-CAG CAC TTC ACG CAT CAG TTT T-3′
	reverse	5′-CCA GCC CGT TGG TGA AAG-3′
iNOS	forward	5′-TGG ATG CAA CCC CAT TGT C-3′
	reverse	5′-CGC TGC CCC AGT TTT TGA T-3′
TNF-α	forward	5′-ATC TTC TCG AAC CCC GAG TGA-3′
	reverse	5′-CGG TTC AGC CAC TGG AGC T-3′
IL-6	forward	5′-CGA GCC CAC CGG GAA CGA AA-3′
	reverse	5′-GGA CCG AAG GCG CTT GTG GAG-3′
IL-1β	forward	5′-CAC GCT CCG GGA CTC ACA GC-3′
	reverse	5′-GGA GAA CAC CAC TTG TTG CTC CA-3′
GAPDH	forward	5′-TGG TAT CGT GGA AGG ACT CA-3′
	reverse	5′-GGG CCA TCG ACA GTC TTC-3′

### Statistical analysis

All conclusions drawn for *ex vivo* studies were derived from at least three independent experiments. Results shown in Figs. 2–5 were means ± SD of three replicates for every real-time PCR reaction within a representative experiment. Differences between treatments and controls were evaluated by two-tailed Student’s *t*-test with *p* < 0.05 or *p* < 0.01 being considered statistically significant or highly significant, respectively. Differences among three or more groups were analyzed by one-way analysis of variance (ANOVA). For *in vivo* studies with ASD patients, the differences between pre-dose and post-dose expression levels of each tested marker were evaluated by two-tailed paired *t*-tests. Grouped markers were analyzed by one-way ANOVA.

## Data Availability

The datasets generated during and/or analyzed during the current study are available from the corresponding author upon reasonable requests.
